# The Association Between the American Heart Association Life’s Essential 8 and Metabolic Syndrome Among Postmenopausal Women: Findings from NHANES 2011–2020

**DOI:** 10.3390/nu17101688

**Published:** 2025-05-15

**Authors:** Harshini Meegaswatte, Andrew J. McKune, Demosthenes B. Panagiotakos, Sukhuntha Osiriphun, Noppol Leksawasdi, Pornchai Rachtanapun, Martin Veysey, Nenad Naumovski, Siraphat Taesuwan

**Affiliations:** 1 Discipline of Nutrition and Dietetics, Faculty of Health, University of Canberra, Canberra, ACT 2617, Australia; harshini.meegaswatte@canberra.edu.au (H.M.); andrew.mckune@canberra.edu.au (A.J.M.); dbpanag@hua.gr (D.B.P.); 2 Functional Foods and Nutrition Research (FFNR) Laboratory, University of Canberra, Canberra, ACT 2617, Australia; 3 University of Canberra Research Institute for Sport and Exercise (UCRISE), University of Canberra, Canberra, ACT 2617, Australia; 4 Discipline of Biokinetics, Exercise and Leisure Sciences, School of Health Science, University of KwaZulu-Natal, Durban 4041, South Africa; 5 Department of Nutrition and Dietetics, School of Health Science and Education, Harokopio University, 17676 Athens, Greece; 6 Faculty of Agro-Industry, Chiang Mai University, Chiang Mai 50100, Thailand; sukhuntha.o@cmu.ac.th (S.O.); noppol.l@cmu.ac.th (N.L.); pornchai.r@cmu.ac.th (P.R.); 7 Center of Excellence in Agro Bio-Circular-Green Industry (Agro BCG), Faculty of Agro-Industry, Chiang Mai University, Chiang Mai 50100, Thailand; 8 School of Medicine and Psychology, Australian National University, Canberra, ACT 2605, Australia; martin.veysey@anu.edu.au

**Keywords:** Life’s Essential 8, cardiovascular disease, metabolic syndrome, cardiovascular health status, menopause, postmenopausal women

## Abstract

**Background:** Postmenopausal women face increased risk of developing metabolic syndrome (MetS) and cardiovascular disease (CVD) due to hormone changes during menopause. Life’s Essential 8 (LE8), a relatively new cardiovascular health assessment index by the American Heart Association, may impact MetS diagnostics and potential treatment strategies. This study investigated the association between LE8 and MetS among postmenopausal women. **Methods**: National Health and Nutrition Examination Survey (NHANES) data were extracted (2011–2020). LE8 comprised eight health behaviours and factors (score of 0–100) classified into low (0–49), moderate (50–79), and high (80–100) cardiovascular health status. MetS was defined as metabolic dysfunctions comprising insulin resistance, central obesity, dyslipidaemia, impaired glucose metabolism, and hypertension. Complex survey-adjusted regression models were used. Effect modification by race and female hormone use were investigated. **Results**: 5402 postmenopausal women were included (mean age: 63.0 y), and 3152 (58.3%) met MetS criteria. LE8 scores for those with and without MetS were 58 ± 13.8 and 70 ± 14.1 (*p* < 0.001), respectively (mean ± SD). Higher LE8 scores were associated with lower odds of having MetS (Odds ratio (OR) for a 10-score increase: 0.53, 95% CI: [0.48, 0.57], *p* < 0.001). Nicotine exposure score was inversely associated with the likelihood of having MetS (0.52 [0.34, 0.80], *p* = 0.022). The association between LE8 status and odds of MetS differed by race (*p-interaction* = 0.01); ‘moderate’ or ‘high’ cardiovascular health status lowered odds of MetS in all races except other/multi-racial. **Conclusions**: The inverse relationship between LE8 scores and MetS suggests that integrating LE8 components into management strategies may help prevent CVD in postmenopausal women.

## 1. Introduction

Metabolic syndrome (MetS) is characterised by a cluster of metabolic dysfunctions comprising insulin resistance, central obesity, atherogenic dyslipidaemia, impaired glucose metabolism, and high blood pressure levels [1,2]. MetS has been consistently linked to an elevated risk of type 2 diabetes mellitus (T2DM) and cardiovascular disease (CVD) [1,2]. Between 1999 and 2018, the prevalence of MetS among adults in the United States (U.S.) increased markedly from 36.2 to 47.3% [3]. This rise has contributed to the heightened incidence of CVD and mortality, particularly among women [4,5].

Menopause, a natural biological process of aging, is marked by the permanent cessation of the menstrual cycle due to the loss of ovarian function [6]. Hormone changes during the menopausal transition contribute to increased abdominal obesity, insulin resistance, dyslipidaemia, vascular dysfunction, and hypertension, thereby elevating the risk of several diseases, including T2DM, metabolic dysfunction-associated fatty liver disease, MetS, and CVD in postmenopausal women [4,7,8,9]. Despite the well-documented adverse effects of the menopausal transition, women in premenopausal, perimenopausal, and postmenopausal stages are often underrepresented in CVD research studies [10].

The American Heart Association’s Life’s Essential 8 (LE8) is a framework developed to assess and promote improvements in cardiovascular health at both the individual and population level [11]. LE8 comprises eight health behaviours and factors: dietary quality, physical activity level, nicotine exposure, sleep health, body mass index (BMI), blood glucose, non-high-density lipoprotein cholesterol (non-HDL), and blood pressure. Each measure is scored and categorised as low (0–49), moderate (50–79), or high (80–100) based on established clinical thresholds, with a higher LE8 score reflecting better cardiovascular health status [11]. LE8 integrates a broader range of behavioural and clinical metrics by accounting for the cumulative and interactive effects of multiple health factors. LE8 offers a nuanced approach to cardiovascular health evaluation across the lifespan by enhancing the ability to monitor and promote heart health in both clinical and public health settings [11]. Previous studies have demonstrated associations between LE8 scores with individual components of MetS, such as blood glucose, hyperlipidaemia, BMI, and blood pressure [12]. Consequently, LE8 scores may also be associated with MetS. The recent introduction of LE8 has generated research interest, and several studies have explored relationships between LE8 and metabolic health in older adults [12,13,14,15,16]. To the best of the author’s knowledge, no studies have examined the association between LE8 and MetS among postmenopausal women. Given that the onset and progression of CVD in postmenopausal women remain underexplored, undetected, and inadequately treated [17], it is crucial to identify the metabolic health changes associated with menopause transition [10]. Applying LE8 to postmenopausal women facilitates improved risk stratification and supports the development of tailored, multidimensional prevention strategies to address their unique cardiovascular health challenges [11]. Therefore, the present study aimed to investigate the association between LE8 and MetS among postmenopausal women within the U.S. population.

## 2. Materials and Methods

### 2.1. Data Source

This cross-sectional study used data from the National Health and Nutrition Examination Survey (NHANES) between 2011 and 2020 (four cycles). This dataset comprises a nationally representative sample of the U.S. population before the COVID-19 pandemic [18].

The NHANES study is conducted by the National Centre for Health Statistics, the Centres for Disease Control and Prevention, as a population-based cross-sectional survey conducted using a multistage stratified sampling design. It includes demographic, socioeconomic, dietary, and health-related questions, physiological measurements, and laboratory examinations. These data provide information on the prevalence and progression of risk factors associated with major diseases [19]. The NHANES surveys have been conducted continually in two-year cycles since 1999 (further details about NHANES can be accessed at https://www.cdc.gov/nchs/nhanes/index.htm). The NHANES protocol was approved by the National Centre for Health Statistics Ethics Review Board, and written informed consent was obtained from all participants.

### 2.2. Study Design and Population

**Figure 1:**
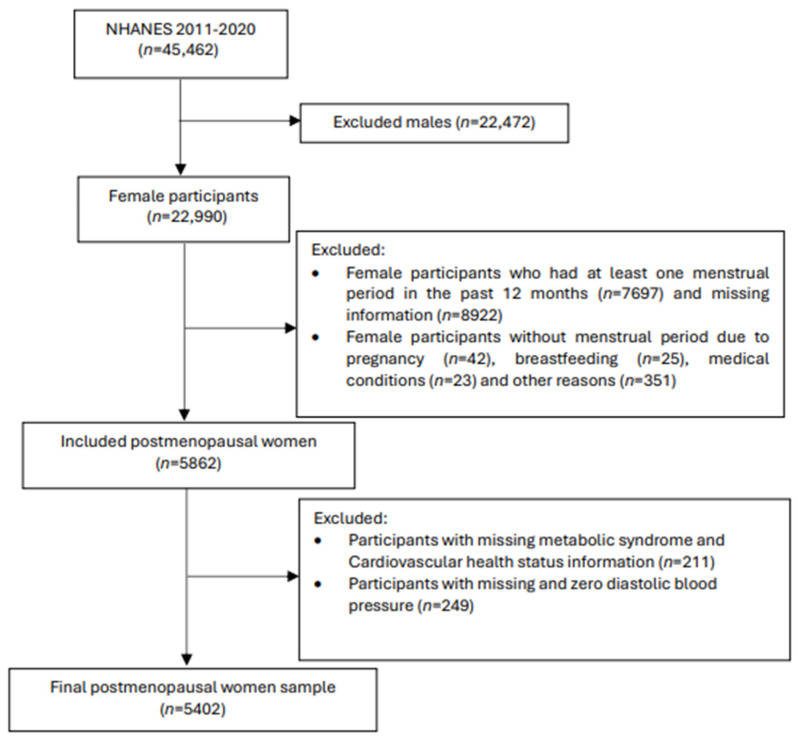
Flow chart of the participant sampling process.

The original 2011–2020 NHANES dataset contained 45,462 participants with the current study including 5862 postmenopausal women. Menopausal status was determined based on the self-reported reproductive health questionnaire. Women were classified as postmenopausal if they did not have a menstrual cycle in the past 12 months and if they had selected ‘hysterectomy’ or ‘menopause/change of life’ as a reason. Participants with missing information required for LE8 and MetS classifications (*n* = 211), and participants with missing or zero diastolic blood pressure (*n* = 249) were excluded. A total of 5402 postmenopausal women with complete data were included in the final analysis (Figure 1).

### 2.3. Measurement of Life’s Essential 8 Score

The components of LE8 include four health behaviours (diet, physical activity, nicotine exposure, and sleep health) and four health factors (BMI, blood lipids, blood glucose, and blood pressure) [11]. Each component has a scoring algorithm from 0 to 100 points, and it was calculated according to the definition outlined by Lloyd-Jones et al. [11]. Supplementary Material provides a comprehensive process for calculating the LE8 scores for each metric within NHANES data, as previously published [20]. Health behaviour and health factor scores were determined based on the unweighted means of four health behaviour components and four health factor components, respectively (0–100), and were categorised into three levels: low (0–49), moderate (50–79), and high (80–100) health behaviour and health factor statuses. Similarly, the LE8 score was calculated using the unweighted average of all eight components, ranging from 0 to 100 points, and classified into three statuses: low (0–49), moderate (50–79), and high cardiovascular health (80–100) [11]. These cutoffs were developed based on a conceptual framework by the American Heart Association, reflecting expert consensus rather than strict empirical thresholds. The cutoffs align with other public health scoring systems and are supported by evidence linking higher LE8 scores to better cardiovascular outcomes.

The diet component of LE8 was assessed using the 2015 version of the Healthy Eating Index (HEI) [21]. HEI-2015 scores were calculated from two days of 24-h dietary food recall data by using the SAS macro from the National Cancer Institute [22]. Self-reported questionnaires were used to collect physical activity, smoking and sleeping behaviours, medical conditions, and medication history. Physical examination data included weight, height, and blood pressure measurements. Biochemical analysis data included blood lipids (total, LDL, and HDL cholesterol, and triglycerides), plasma glucose, and haemoglobin A1c (HbA1c).

### 2.4. Measurements and Criteria of MetS

Participants were classified as having MetS if they had at least three out of five conditions: (1) increased waist circumference (≥88 cm in women and ≥102 cm in men), (2) elevated triglycerides (≥150 mg/dL) or treatment for high triglycerides, (3) reduced HDL cholesterol (<40 mg/dL in men and <50 mg/dL in women) or medication for low HDL cholesterol levels, (4) high blood pressure (systolic blood pressure ≥130 mmHg, diastolic blood pressure ≥ 85 mmHg, or both) or antihypertensive medication, and (5) elevated fasting glucose levels (≥100 mg/dL) or treatment for hyperglycaemia [1,2].

### 2.5. Other Measurements

Demographic variables included age (years), race (Mexican American, other Hispanic, non-Hispanic white, non-Hispanic black, and other race including multi-racial), marital status (married or living with partner, widowed/Divorced/Separated or never married), education level (less than 9th grade, 9–11th grade, high school graduate or some college degree, and college graduate or above), and family income level (measured as the ratio of family income to poverty). Alcohol intake (presence or absence) and the average amount of alcohol consumed per day were also included in the analysis. 

### 2.6. Statistical Analysis

Reflecting the complex sampling design of NHANES, all analyses were adjusted for sampling weights, clustering, and stratification to ensure nationally representative results. To establish the correct estimation of national population parameters over the combined study period, new sampling weights were calculated for the 2011–2020 cycles according to the NHANES guidelines. A univariate analysis was conducted to examine all variables for errors, outliers, and deviations from a normal distribution. Continuous variables with a normal distribution were expressed as mean ± standard deviation (SD) and non-normal data were expressed as median and interquartile range. Categorical variables were expressed as counts and percentages. Complex survey logistic regression models were implemented to determine the odds ratio (OR) and 95% confidence intervals (CI) for the associations of LE8 scores and statuses with MetS. Two models were used: crude models not adjusted for any covariate and full models adjusted for age, race, marital status, education level, the ratio of family income to poverty, alcohol drinking status, and average alcohol consumed per day. Furthermore, the associations of the health behaviour or health factor scores (as categorical variables) with MetS were investigated using the complex survey logistic regression. Two models were used in this analysis; Model 1 was adjusted for age, race, marital status, education level, the ratio of family income to poverty, alcohol drinking status, and average alcohol consumed per day. Model 2 was further adjusted for health behaviour or health factor components that were not used in the respective models. Subgroup analysis was carried out to explore effect modification by race and female hormone use. All analyses were performed using IBM SPSS Statistics for Windows (v29, IBM Corp, Armonk, NY, USA), and *p* < 0.05 was used to indicate statistical significance in two-sided statistical testing.

## 3. Results

### 3.1. Baseline Characteristics

A total of 5402 postmenopausal women, with a weighted mean age of 63 ± 10 years, were included in the analyses. Among them, 3152 (58.3%) were categorised as living with MetS. Baseline characteristics of the study sample are summarized in Table 1. Postmenopausal women with MetS were more likely to be older, non-Hispanic white, married or living with a partner, and to have a lower education level, higher BMI, and higher waist circumference when compared to those without MetS. The mean LE8 score, health behaviour score, and health factor score of postmenopausal women with MetS were lower than those of the postmenopausal women without MetS (58 vs. 70 (*p* < 0.001), 65 vs. 71 (*p* < 0.001), and 51 vs. 69 (*p* < 0.001), respectively). Mean scores of all LE8 components were also lower in participants with MetS (*p* < 0.05). Only 165 (5.6%) postmenopausal women with MetS had a ‘high’ cardiovascular health status, compared to 621 (29%) without MetS (Table 1).

### 3.2. Relationships Between Life’s Essential 8 and Metabolic Syndrome in Postmenopausal Women

Complex survey logistic regression analysis indicated that the odds of having MetS decreased with increasing LE8 scores (Table 2). Specifically, a 10-unit score increase in LE8 was associated with a 47% decrease in the odds of having MetS in postmenopausal women (OR: 0.53, 95% CI: [0.48–0.57]). Moderate and high cardiovascular health statuses were associated with a 63% and 93% reduction in the odds of MetS, respectively, compared to the low cardiovascular health status (*p* < 0.001).

The LE8 scores can be separated into two component scores, the health behaviour and health factor scores. Health behaviour scores combined scores from diet, physical activity, nicotine exposure, and sleep health components. Health factor scores combined scores from BMI, blood lipids, blood glucose, and blood pressure components. Among postmenopausal women, both health behaviour and health factor scores were inversely associated with MetS (0.87, [0.84–0.92], and 0.54, [0.51–0.60], respectively). Graded associations were also observed for both health behaviour and health factor statuses, such that ‘moderate’ and ‘high’ (vs. low) statuses were associated with 44–76% and 50–95% lower odds of MetS, respectively (*p* < 0.001; Table 2). A sensitivity analysis using race-specific waist circumference cutoffs was conducted, and the results remained consistent with the primary findings (Supplementary Table S2).

### 3.3. Decomposition of Life’s Essential 8 and Metabolic Syndrome in Postmenopausal Women

To identify key components that influence the relationships between LE8 and MetS, each of the eight components of LE8 was separately investigated. Nicotine exposure was the only health behaviour that was associated with MetS, with never-smokers showing a 48% reduction in the odds of MetS compared to current smokers (OR: 0.52, 95% CI: [0.34–0.80]; Table 3).

Each of the four health factor scores was independently associated with MetS (*p* < 0.001), with higher scores being generally associated with lower odds of MetS. For example, a BMI score of 100 (vs. 0) was associated with 81% decreased odds of MetS (0.19, [0.11–0.31]). Blood glucose scores of 60 and 100 (vs. 0) were also negatively associated with MetS (0.10, [0.021–0.47] and 0.02, [0.001–0.10], respectively). Higher blood lipid scores exhibited inverse associations with MetS, except for the score of 80, which showed a positive association. It should be noted that there is a high number of MetS participants in this score category (962 out of 982 people). This pattern was also observed for blood pressure scores, where people who used antihypertensive medication were positively associated with increased odds of MetS despite having normal blood pressure levels (score of 80). On the other hand, people who truly had normal blood pressure (score of 100) showed a negative association with MetS. These inconsistent results are plausible because MetS comprised five criteria, and participants who appeared normal by one criterion may still be classified as having MetS by the other criteria.

To provide additional clinical and epidemiological insights, the associations between LE8 and each individual component of MetS were examined. LE8 scores were found to be associated with all MetS components except triglycerides (Supplementary Table S3).

### 3.4. Effect Modification by Race and Female Hormone Usage

#### 3.4.1. Effect Modification by Race

**Figure 2:**
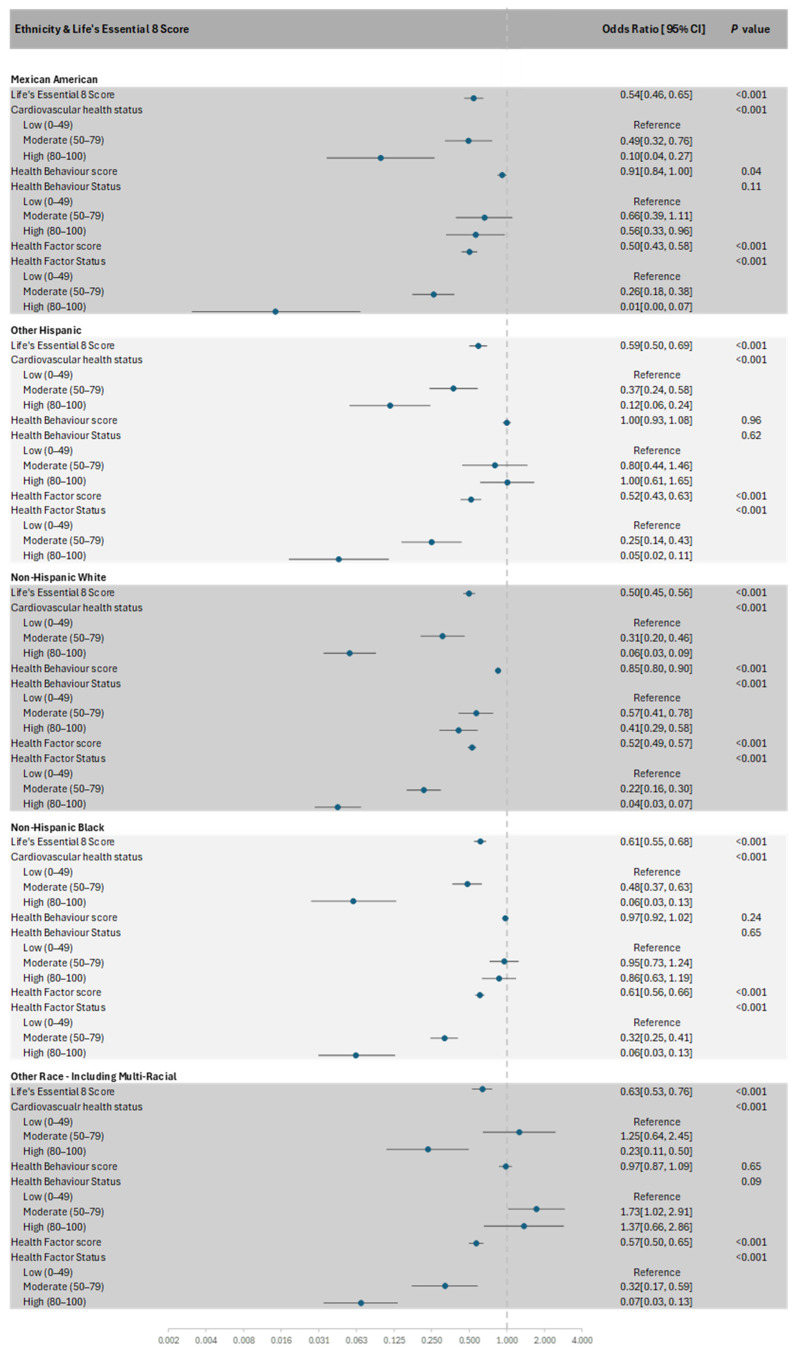
Forest plot of the subgroup analysis of Life’s Essential 8 scores with the presence of MetS in each race.

Race modified the relationships between cardiovascular health status and MetS (*p-interaction* = 0.01), such that a ‘moderate’ or ‘high’ cardiovascular health status was associated with lower odds of MetS in all races except for other races/multi-racial, whose beneficial association was observed only for the ‘high’ cardiovascular health status (Figure 2).

Health behaviour scores were inversely associated with MetS in Mexican Americans and non-Hispanic Whites (*p-interaction* = 0.01). When the scores were categorised into health behaviour status, an inverse association with MetS was only observed in non-Hispanic Whites (*p-interaction* = 0.003). No significant interactions were observed between race and cardiovascular health scores (*p-interaction* = 0.07), health factor scores (*p-interaction* = 0.09), and health factor status (*p-interaction* = 0.60).

#### 3.4.2. Effect Modification by Female Hormone Usage

In a fully adjusted analysis, female hormone usage (in the form of hormonal supplementation) did not interact with any of the LE8 measures: LE8 scores (*p-interaction* = 0.57), cardiovascular health status (*p-interaction* = 0.38), health behaviour score (*p-interaction* = 0.51), health behaviour status (*p-interaction* = 0.11), health factor score (*p-interaction* = 0.56), and health factor status (*p-interaction* = 0.90; Supplementary Table S1).

## 4. Discussion

This study examined the relationship between the American Heart Association LE8 and the prevalence of MetS among postmenopausal women using data from the 2011-2020 NHANES. The findings indicated that overall LE8 scores, as well as health behaviour and health factor scores and statuses were inversely related to MetS in this population. Among the four health behaviour components of LE8, only the nicotine exposure score was negatively associated with the likelihood of MetS, whereas all four health factor components demonstrated significant associations with MetS. Subgroup analysis revealed that the associations between cardiovascular health status (derived from LE8 scores), health behaviour scores, and health behaviour status with MetS were evident only in certain racial groups. In contrast, the associations between health factors and MetS were consistent across all racial groups. To the best of our knowledge, this is the first study to explore the associations of LE8 scores and cardiovascular health status with MetS prevalence specifically in postmenopausal women. Previous cross-sectional studies utilizing NHANES data have investigated the association between LE8 and the prevalence of MetS in the general population, demonstrating that higher LE8 scores were significantly associated with lower odds of MetS [12,13,14,15]. Yang et al. conducted a cross-sectional study using the NHANES dataset, which indicated an inverse relationship between LE8 scores and the prevalence of metabolic dysfunction in the general adult population [16].

The primary findings of this study suggested that even a modest increase in the LE8 score was associated with a substantial reduction in the odds of having MetS in postmenopausal women. These robust effect estimates imply that MetS can be mitigated in postmenopausal women through lifestyle modifications and increased physical activity targeting all LE8 components. Improvements in four key health behaviours, i.e., diet, sleep, physical activity, and smoking, were associated with 13% reduced odds of MetS for each 10-unit increase in the behavioural score. Furthermore, each 10-unit increase in health factor scores (BMI, blood glucose, blood pressure, and lipids) was associated with a 46% lower likelihood of MetS. These results underscore the importance of lifestyle changes leading to physiological improvements as a strategy for preventing MetS during the menopausal transition, a period characterized by hormonal changes that elevate the risk of abdominal obesity, dyslipidaemia, hyperglycaemia, insulin resistance, and hypertension [4,10,23].

In the decomposition analysis of the LE8 score, avoidance of nicotine exposure was strongly and inversely associated with MetS. Postmenopausal women who never smoked had 48% lower odds of MetS compared to current smokers. The other health behaviours (sleep health, physical activity, and diet) did not show significant associations with MetS in this study. The absence of associations may be attributed to misclassification, given the imprecise nature of these human behaviours. In contrast, smoking behaviour is clearly defined and can be accurately measured, facilitating the detection of the association. Within the scientific literature, smoking is well established as a strong risk factor for the development of CVD. Previous studies have indicated that poor sleep quality in postmenopausal women is associated with poor cardiovascular health [24], insulin resistance [25], and increased current and future risks of MetS and some of its components [26,27,28]. Similarly, the HEI-2015 diet score is related to MetS in postmenopausal women [29,30,31].

Hormonal changes during menopause significantly influence body fat distribution, contributing to weight gain and abdominal obesity, thereby increasing insulin resistance and the risk of T2DM in postmenopausal women [32]. The present study demonstrated that a lower BMI or HbA1C was associated with reduced odds of MetS, underscoring the importance of weight management and blood glucose monitoring during this transitional phase. Additionally, the reduction in estrogen levels during menopause is frequently linked to an elevated risk of dyslipidaemia and may expedite vascular aging [4,11,12,14]. This process encompasses endothelial cell dysfunction, vascular remodelling, increased vascular stiffness, and inflammation [33]. The acceleration of vascular aging in postmenopausal women contributes to heightened risks of hypertension and CVD [34,35]. The study further reveals that lower levels of non-HDL cholesterol or blood pressure levels (in the absence of medication) are generally associated with MetS protection in postmenopausal women.

Race differences influenced the relationships between cardiovascular health status, health behaviour score, and health behaviour status with MetS, with the strongest associations generally observed in non-Hispanic Whites. However, this pattern may differ among specific Asian subpopulations and evolve over time, particularly as immigrant groups undergo acculturation and adopt Western lifestyle behaviours associated with increased cardiovascular risk. Although research in this domain remains relatively limited, several studies evaluating the association of health behaviours across different races and ethnicities align with these findings [24,36,37,38].

A notable strength of this study is its novel investigation of the association between the LE8 scores and MetS using a large, nationally representative sample of postmenopausal women. This research has a potential to inform guidelines for the prevention and management of MetS and cardiovascular issues in postmenopausal women. However, the primary limitation is the inability to establish causality due to the cross-sectional nature of the NHANES data. Unknown or residual confounding factors may have influenced the study’s outcome. Misclassification in health behaviour components could affect the consistency of the results. Data on dietary, physical activity, and sleep health were collected through self-reported questionnaires rather than more accurate and reliable objective methods. Although the hormonal changes during menopause are associated with sleep quality and MetS in postmenopausal women [39,40], this aspect is not addressed in the LE8. These factors may lead to misclassification and potentially impact the validity of the observed associations between health behaviour scores and MetS in postmenopausal women. Additionally, the overlap between health factors in the LE8 and the diagnostic criteria of MetS may result in inconsistent patterns in the findings and collinearity, which may have contributed to an overestimation of associations, particularly for the health factor components. However, given that health behaviour scores remained significantly and inversely associated with MetS, the protective direction of association between LE8 and MetS is likely to be true. Another potential shortcoming of this study is the use of uniform waist circumference cutoffs, rather than race-specific thresholds, which could have impacted the accuracy of MetS classification.

## 5. Conclusions

In conclusion, the findings underscore the significant inverse associations between cardiovascular health, as assessed by LE8 scores, and the prevalence of MetS in U.S. postmenopausal women. While all four health factor components of LE8 were strongly linked to MetS, only nicotine exposure among the health behaviour components demonstrated a significant association, highlighting the critical impact of cigarette smoking on cardiometabolic health. These associations varied across ethnic groups, emphasizing the necessity for tailored interventions to enhance cardiovascular health and mitigate MetS risk. Future research should investigate the underlying mechanisms driving these disparities and evaluate targeted strategies to improve health outcomes in diverse populations.

## Figures and Tables

**Table 1:** Baseline characteristics of the NHANES study sample ^a^.

Variables	Total (*n* = 5402)	Metabolic Syndrome (*n* = 3152)	No Metabolic Syndrome (*n* = 2250)	*p* Value
Age (year)	63 ± 10	65 ± 10	61 ± 10	<0.001
Race/Hispanic origin, *n* (%)				
Mexican American	574 (4.4)	367 (5.0)	207 (3.7)	0.001
Other Hispanic	653 (5.2)	398 (5.7)	255 (4.7)	0.033
Non-Hispanic White	2245 (73.5)	1262 (71.1)	983 (76.3)	<0.001
Non-Hispanic Black	1301 (10.2)	796 (11.3)	505 (8.9)	<0.001
Other Race—Including Multi-Racial	629 (6.7)	329 (7.0)	300 (6.3)	0.414
Marital status, *n* (%)				
Married/Living with Partner	2653 (57.8)	1481 (55.2)	1172 (60.8)	0.007
Widowed/Divorced/Separated	2297 (36.7)	1417 (39.2)	880 (33.7)	0.004
Never married	446 (5.6)	250 (5.6)	196 (5.5)	0.93
Ratio of family income to poverty	3.08 ± 1.61	2.85 ± 1.59	3.35 ± 1.60	<0.001
Education levels, *n* (%)				
Less than 9th grade	574 (5.0)	409 (6.5)	165 (3.2)	<0.001
9–11th grade	661 (8.6)	433 (10.2)	228 (6.6)	<0.001
High school graduate	1319 (25.9)	807 (29.1)	512 (22.3)	<0.001
Some college	1728 (33.7)	989 (33.9)	739 (33.4)	0.789
College graduate or above	1117 (26.8)	512 (20.3)	605 (34.4)	<0.001
Body mass index (Kgm^−2^)	29.79 ± 7.17	31.77 ± 7.19	27.50 ± 6.42	<0.001
Waist circumference (cm)	100.27 ± 15.49	105.42 ± 14.70	94.47 ± 14.25	<0.001
Average alcohol consumed (drinks per day)	0.258 ± 0.63	0.179 ± 0.48	0.350 ± 0.76	<0.001
Life’s Essential 8 score ^b^	63.74 ± 15.19	58.08 ± 13.76	70.34 ± 14.07	<0.001
Health behaviour score ^b^	67.95 ± 21.13	65.02 ± 21.48	71.37 ± 20.18	<0.001
Healthy Eating Index-2015 diet score	49.30 ± 31.21	47.41 ± 30.92	51.48 ± 31.39	0.002
Physical activity score	62.63 ± 45.19	56.30 ± 46.71	70.01 ± 42.19	<0.001
Nicotine exposure score	78.60 ± 39.23	77.51 ± 40.01	79.83 ± 38.31	0.184
Sleep health score	83.58 ± 24.60	82.13 ± 25.44	85.26 ± 23.47	0.004
Health factor score ^b^	59.70 ± 18.81	51.38 ± 16.10	69.38 ± 17.03	<0.001
Body mass index score	56.49 ± 34.42	46.65 ± 32.92	67.87 ± 32.55	<0.001
Blood lipids score	58.83 ± 29.36	55.97 ± 29.16	62.22 ± 29.24	<0.001
Blood glucose score	70.01 ± 26.69	58.65 ± 25.29	83.38 ± 21.62	<0.001
Blood pressure score	54.19 ± 34.52	45.03 ± 31.91	64.88 ± 34.39	<0.001
Cardiovascular Health, *n* (%) ^c^				
Low	836 (17.9)	674 (26.0)	162 (8.5)	<0.001
Moderate	3780 (65.7)	2313 (68.4)	1467 (62.5)	0.007
High	786 (16.4)	165 (5.6)	621 (29.0)	<0.001

^a^ Continuous variables with normal distribution were expressed as mean ± standard deviation. Non-normal data (average alcohol consumed) were expressed as median interquartile range, and categorical variables were presented as numbers (percentage). ^b^ Life’s Essential 8 scores, health behaviour scores, and health factor scores range from 0–100. ^c^ Cardiovascular health was defined using Life’s Essential 8 scores as follows: low, 0 to 49; moderate, 50 to 79: and high, 80 to 100.

**Table 2:** Associations of the Life’s Essential 8 scores with metabolic syndrome in postmenopausal women in 2011–2020 NHANES study.

	Crude Model ^a^		Full Model ^b^	
	Odds Ratio (95% CI)	*p* Value	Odds Ratio (95% CI)	*p* Value
Life’s Essential 8 score (per 10 units)	0.53 (0.50–0.57)	<0.001	0.53 (0.48–0.57)	<0.001
Cardiovascular health status				<0.001
Low (0–49)	1 (Reference)		1 (Reference)	
Moderate (50–79)	0.36 (0.28–0.45)	<0.001	0.37 (0.28–0.50)	<0.001
High (80–100)	0.06 (0.05–0.08)	<0.001	0.07 (0.05–0.10)	<0.001
Health behaviour score (per 10 units)	0.86 (0.83–0.90)	<0.001	0.87 (0.84–0.92)	<0.001
Health behaviour status				<0.001
Low (0–49)	1 (Reference)		1 (Reference)	
Moderate (50–79)	0.66 (0.54–0.81)	<0.001	0.66 (0.51–0.85)	<0.001
High (80–100)	0.46 (0.37–0.57)	<0.001	0.50 (0.38–0.67)	<0.001
Health factor score (per 10 units)	0.52 (0.50–0.55)	<0.001	0.54 (0.51–0.60)	<0.001
Health factor status				<0.001
Low (0–49)	1 (Reference)		1 (Reference)	
Moderate (50–79)	0.23 (0.19–0.27)	<0.001	0.24 (0.19–0.29)	<0.001
High (80–100)	0.04 (0.03–0.06)	<0.001	0.05 (0.03–0.07)	<0.001

^a^ Crude Model: Not adjusted for any covariate. ^b^ Full Model: Adjusted for race, marital status, education level, age, ratio of family income to poverty, alcohol drinking status, and average alcohol consumed per day.

**Table 3:** Associations of Life’s Essential 8 components with metabolic syndrome in postmenopausal women in 2011–2020 NHANES.

	Model 1		Model 2	
	OR (95% CI)	*p* Value	OR (95% CI)	*p* Value
Health behaviour scores:				
Healthy eating index-2015 diet score (percentile)		0.076		0.852
0 (1st–24th)	1 (Reference)		1 (Reference)	
25 (25th–49th)	0.97 (0.73–1.29)		0.89 (0.59–1.36)	
50 (50th–74th)	0.86 (0.69–1.07)		0.83 (0.55–1.24)	
80 (75th–94th)	0.84 (0.65–1.08)		0.98 (0.63–1.51)	
100 (≥95th)	0.61 (0.42–0.86)		0.85 (0.49–1.48)	
Physical activity score (min of moderate-to-vigorous physical activity per week)		<0.001		0.096
0 (0 min)	1 (Reference)		0 (Reference)	
20 (1–29 min)	0.58 (0.30–1.12)		0.44 (0.10–1.86)	
40 (30–59 min)	0.66 (0.38–1.15)		0.83 (0.29–2.32)	
60 (60–89 min)	0.54 (0.35–0.81)		0.44 (0.23–0.87)	
80 (90–119 min)	1.19 (0.64–2.21)		1.53 (0.58–4.04)	
90 (120–149 min)	0.58 (0.37–0.89)		1.07 (0.57–2.01)	
100 (≥150 min)	0.65 (0.52–0.80)		1.12 (0.77–1.63)	
Nicotine exposure score		0.071		0.022
0 (Current smoker)	1 (Reference)		1 (Reference)	
25 (quit < 1 yr)	0.66 (0.18–2.42)		0.69 (0.10–4.69)	
50 (quit 1–<5 yrs)	0.45 (0.16–1.23)		0.22 (0.03–1.53)	
75 (quit ≥5 yrs)	0.76 (0.45–1.29)		0.59 (0.31–1.11)	
100 (Never Smoker)	0.65 (0.48–0.87)		0.52 (0.34–0.80)	
Sleep health score		0.009		0.34
0 (<4 h)	1 (Reference)		1 (Reference)	
20 (4–<5 h)	0.96 (0.39–2.38)		1.08 (0.32–3.60)	
40 (5–<6 or ≥10 h)	1.31 (0.58–2.98)		0.89 (0.27–2.99)	
70 (6–<7 h)	0.75 (0.33–1.73)		0.60 (0.17–2.18)	
90 (9–<10 h)	0.95 (0.41–2.24)		0.58 (0.17–1.95)	
100 (7–<9 h)	0.78 (0.36–1.66)		0.78 (0.22–2.77)	
Health factor scores:				
Body mass index score (kg/m^2^)		<0.001		<0.001
0 (>40)	1 (Reference)		1 (Reference)	
15 (35.0–39.9)	0.76 (0.52–1.11)		1.09 (0.60–1.97)	
30 (30.0–34.9)	0.57 (0.39–0.81)		0.89 (0.49–1.61)	
70 (25.0–29.9)	0.33 (0.23–0.46)		0.71 (0.42–1.19)	
100 (<25)	0.13 (0.09–0.18)		0.19 (0.11–0.31)	
Blood lipids score (non–HDL cholesterol level)		<0.001		<0.001
0 (>220 or 190–219 mg/dL and using medication)	1 (Reference)		1 (Reference)	
20 (190–219 or 160–189 mg/dL and using medication)	0.57 (0.36–0.91)		0.30 (0.16–0.56)	
40 (160–189 or 130–159 mg/dL and using medication)	0.43 (0.30–0.62)		0.35 (0.19–0.62)	
60 (130–159 mg/dL)	0.16 (0.11–0.24)		0.10 (0.05–0.19)	
80 (<130 mg/dL and using medication)	8.08 (4.36–14.98)		12.65 (5.15–31.12)	
100 (<130 mg/dL)	0.13 (0.09–0.21)		0.08 (0.05–0.14)	
Blood glucose score		<0.001		<0.001
0 (Diabetes with HbA1C ≥10.0)	1 (Reference)		1 (Reference)	
10 (Diabetes with HbA1C 9.0–9.9)	0.78 (0.16–3.88)		0.43 (0.05–4.06)	
20 (Diabetes with HbA1C 8.0–8.9)	1.21 (0.33–4.45)		1.22 (0.17–8.66)	
30 (Diabetes with HbA1C 7.0–7.9)	0.38 (0.12–1.18)		0.34 (0.06–1.96)	
40 (Diabetes with HbA1C <7.0)	0.31 (0.12–0.80)		0.23 (0.05–1.17)	
60 (No diabetes with HbA1C 5.7–7.0)	0.07 (0.03–0.18)		0.10 (0.02–0.47)	
100 (No diabetes with HbA1C <5.7)	0.02 (0.01–0.05)		0.02 (0.00–0.10)	
Blood pressure score		<0.001		<0.001
0 (≥160 or ≥100 mmHg)	1 (Reference)		1 (Reference)	
5 (140–150 or 90–99 mmHg and using medication)	1.86 (1.26–2.75)		2.08 (1.13–3.82)	
25 (140–150 or 90–99 mmHg)	1.00 (0.59–1.69)		1.62 (0.76–3.46)	
30 (130–139 or 80–89 mmHg and using medication)	1.61 (1.06–2.45)		1.49 (0.81–2.76)	
50 (130–139 or 80–89 mmHg)	0.54 (0.38–0.77)		0.63 (0.36–1.11)	
55 (120–129/<90 mmHg and using medication)	2.83 (1.70–4.73)		2.09 (0.90–4.87)	
75 (120–129/<90 mmHg)	0.32 (0.20–0.51)		0.27 (0.14–0.54)	
80 (<120/<80 mmHg and using medication)	2.81 (1.79–4.40)		2.81 (1.52–5.21)	
100 (<120/<80 mmHg)	0.20 (0.14–0.29)		0.17 (0.09–0.32)	

Model 1: Adjusted for race, marital status, education level, age, ratio of family income to poverty, alcohol drinking status, and average alcohol consumed per day. Model 2: Adjusted for Model 1 variables, plus Life’s Essential 8 components that were not used in the respective model. Abbreviations: HbA1C, glycosylated haemoglobin.

## Data Availability

The data presented in this study are from publicly available data in NHANES available resources at https://www.cdc.gov/nchs/nhanes/index.htm and other additional data are available in the article and Supplementary Material.

## Supplementary Materials

The following supporting information can be downloaded at: https://www.mdpi.com/article/10.3390/nu17101688/s1, Table S1: Subgroup analysis of the Interaction of the usage of female hormones and Life’s Essential 8 scores with the presence of metabolic syndrome; Table S2: Associations of the Life’s Essential 8 scores with metabolic syndrome (with race/ethnicity-specific waist circumference cut-offs) in postmenopausal women in 2011–2020 NHANES study; Table S3: Associations of metabolic syndrome components with Life’s Essential 8 scores in postmenopausal women in 2011–2020 NHANES; Table S4: STROBE-nut: An extension of the STROBE statement for nutritional epidemiology. Reference [41] is cited in the supplementary materials.

## Abbreviations

The following abbreviations are used in this manuscript

BMI	Body mass index
CI	Confidence intervals
CVD	Cardiovascular disease
HbA1c	Haemoglobin A1c
HEI	Healthy Eating Index
LE8	Life’s Essential 8
MetS	Metabolic syndrome
non-HDL	Non-high-density lipoprotein cholesterol
OR	Odds ratio
SD	Standard deviation
NHANES	The National Health and Nutrition Examination Survey
T2DM	Type 2 diabetes mellitus
